# Current News

**Published:** 1900-06

**Authors:** 


### Current News.

### RESOLUTIONS REGARDING DR. J. N. CROUSE AND THE DENTAL PROTECTIVE ASSOCIATION.

The following resolutions were adopted by the Illinois State Dental Society, at Springfield, May 9, 1900:

In view of the recent activity on the part of the International Tooth Crown Company, their agents and allies, both in and out of the profession, and the appearance at the same time in some of the dental journals of articles reflecting on the management and tending to destroy confidence in the Dental Association, the Illinois State Dental Society, in the thirty-sixth annual meeting assembled, deems it most opportune at this time to again put on record its confidence in and loyalty to the Association and its management, which has made of it such a wall of protection to the entire profession.

This Society desires also in the most emphatic manner to express its confidence in the personal integrity of Dr. J. N. Crouse, and hereby records its deep appreciation of the unselfish personal sacrifices that he has so freely made, for so many years, for the good of the cause.

This Society considers the cause of the Dental Protective Association the cause of the profession, and it condemns as inimical to the interests of the entire profession the circulation of slanders by travelling men or the publication of articles that are calculated to weaken the hands of those who are fighting our battles and to put weapons into the hands of our enemies. And this Society hereby calls upon all those who have the good of the profession at heart to give the Dental Protective Association a most cordial and unqualified support, and counsels them to show in no uncertain way their utter disapproval of all those who give aid and comfort to the common enemy.

C. B. Rohland,
E. K. Blair,
J. G. Reid,
*Committee.*

---

### NATIONAL DENTAL ASSOCIATION, SECTION III.

At the last meeting of the National Dental Association, it was decided by Section III. to make the work of this section a feature of the meeting. To this end it has been arranged to hold the meetings of this section at such times as will not interfere with the general sessions. All papers upon the subjects embraced in this section will be read in these meetings excepting two or three, which, from their general interest, have been selected by the committee for presentation to the general body.

A suitable room will be provided and the programme for each meeting duly announced. Some good papers are promised.

It was further decided to hold clinics. These will comprise operations upon patients and demonstrations upon casts, models, etc. It is desired that every one who has anything new, original, and helpful will bring or send his appliances, models, and illustrations. While new appliances may be shown subject to the provisions of the constitution, nothing can be offered for sale. Suitable provision will be made for the carrying out of these clinics.

Let every one interested in this section who has anything to offer communicate at once with

Thos. E. Weeks, *Chairman,*
Dayton Building, Minneapolis, Minn.,
Jno. J. Hart, *Secretary,*
118 West Fifty-fifth Street, New York,
or Thos. P. Hinman, *Chairman Clinic Committee,*
Atlanta, Ga.

### MASSACHUSETTS DENTAL SOCIETY.

The thirty-sixth annual meeting of the Massachusetts Dental Society will be held in the American House, Hanover Street, Boston, on Wednesday and Thursday, June 6 and 7, 1900. The meeting, clinics, and exhibits will all be held under one roof. An especial clinic is to be given on Porcelain Work by a prominent Philadelphia dentist. Good talent is also promised both for papers and other clinics. The exhibits will also be extensive. The hotel will give special rates and good accommodations. It is hoped that a large number will be in attendance, and a cordial invitation is extended to all reputable dentists to be present.

EDGAR O. KINSMAN,

*Secretary.*

CAMBRIDGE, MASS.

---

### INTERNATIONAL DENTAL CONGRESS.

At the International Dental Congress, to be held in Paris, France, August 8 to 14, 1900, the following papers will be read by the gentlemen named:

A. K. Fort, D.D.S., Atlanta, Ga., "The Influence of the Saliva on Bacterial Growth in the Mouth;" W. A. Price, D.D.S., Cleveland, Ohio, "The Science of Dental Radiography" (illustrated); Richard Grady, M.D., D.D.S., Baltimore, Md., "Instructing our Patients in the Care of the Mouth and Teeth;" R. R. Andrews, A.M., D.D.S., Cambridge, Mass., "The Development of the Enamel;" Geo. W. Cook, D.D.S., Chicago, Ill., "A Bacteriological Study of Pyorrhœa Alveolaris;" C. S. Case, M.D., D.D.S., Chicago, Ill., "Important Principles in Dento-Facial Orthopædia;" R. H. Hofheinz, D.D.S., Rochester, N. Y., "Our Preliminary Educational Deficiencies;" E. H. Angle, M.D., D.D.S., St. Louis, Mo., "The American Type of Dento-Facial Deformity;" J. E. Hinkins, D.D.S., Chicago, Ill., "The Chemical Action of Cements in the Mouth;" I. N. Broomell, D.D.S., Philadelphia, Pa., "The Source of Nutrition of the Dental Pulp;" T. W. Brophy, M.D., D.D.S., LL.D., Chicago, Ill., "Surgical Treatment of Palatal Defects;" W. C. Barrett, M.D., D.D.S., Buffalo, N. Y., "International Dental Ethics;" B. Holly Smith, M.D., D.D.S., Baltimore, Md., will open the discussion on "Education;" Jonathan Taft, A.M., M.D., D.D.S., Cincinnati, Ohio, "Dental History;" A. W. Harlan, A.M., M.D., D.D.S., Chicago, Ill., "Pulp Digestion;" E. R. Warner, M.D., D.D.S., Denver, Col., "Some phases of Mummification."

It is expected that a few additions may be made to this list.

The following gentlemen will give clinics:

W. V.-B. Ames, D.D.S., Chicago, Ill., "Some Possibilities of New Process Oxyphosphate of Copper;" Gordon White, D.D.S., Nashville, Tenn., "A Compound Filling, using in the Cavity Tin, Abbey's Non-Cohesive Gold, and Nickold's Cohesive Gold;" Joseph Head, M.D., D.D.S., Philadelphia, Pa., "Porcelain Inlays;" Alfred Owre, M.D., D.D.S., Minneapolis, Minn., will prepare a step cavity in an incisor or bicuspid, and fill same with DeTrey's Crystal Mat Gold (Solila); Joseph W. Wassall, M.D., D.D.S., Chicago, Ill., "The Treatment of Septic Pulpless Teeth;" Hart J. Goslee, D.D.S., Chicago, Ill., "Porcelain Crowns and Bridge-Work;" Robert Good, D.D.S., Chicago, Ill., "Porcelain Bridge-Work;" V. H. Jackson, M.D., D.D.S., New York, "Jackson's System of constructing Appliances for the Correction of Irregularities of the Teeth;" Levitt E. Custer, D.D.S., Dayton, Ohio, "The Electric Oven, and Electric Gold Annealer;" W. E. Griswold, D.D.S., Denver, Col., "A Removable Crown for the Support of Saddle Plates or Bridges;" E. K. Wedelstaedt, D.D.S., St. Paul, Minn., "Gold Filling,—Mesio-Occlusal Cavity in Upper First Molar," demonstrating Dr. C. V. Black's method of (1) cavity preparation, (2) extension for prevention, (3) occlusal anchorage, (4) the use of annealed and unannealed gold, (5) method of finishing (using the Black saw and finishing files), (6) proper contact, also (7) the scientific application of the rubber dam, and (8) the Wedelstaedt system of measurement, and its application to cavities in the human teeth; Frank Holland, M.D., D.D.S., Atlanta, Ga., "Cohesive Gold Filling;" T. W. Brophy, M.D., D.D.S., LL.D., Chicago, Ill., "Surgical Treatment of Congenital Cleft Palate."

There are three or four additional clinicians to be heard from.

A. W. Harlan, *Chairman.*
W. E. Griswold, *Secretary.*

### WISCONSIN STATE DENTAL SOCIETY.

The thirtieth annual meeting of the Wisconsin State Dental Society will be held at La Crosse, Wis., July 17, 18, and 19, 1900. A cordial invitation is extended to all members of the profession to be present.

W. H. Mueller.
*Secretary.*

### CALIFORNIA STATE DENTAL ASSOCIATION.

The California State Dental Association will hold its annual meeting in San Francisco, June 19, 1900, continuing four days.

W. B. King,
*Secretary.*

### CENTRAL DENTAL ASSOCIATION OF NORTHERN NEW JERSEY.

At the annual meeting of the Central Dental Association the following officers were elected for the ensuing year:

President, H. S. Sutphen, Newark; Vice-President, F. G. Gregory, Newark; Secretary, N. M. Chitterling, Bloomfield; Treasurer, Chas. A. Meeker, Newark.

*Executive Committee.*—J. S. Vinson, Newark; F. L. Hindle, New Brunswick; C. W. Hoblitzell, Jersey City; J. W. Fisher, East Orange; P. G. Voegtlen, Madison.

Nelson M. Chitterling,
*Secretary.*

### COLORADO STATE DENTAL ASSOCIATION.

The fourteenth annual meeting of the Colorado State Dental Association will be held in Boulder, Col., Tuesday, Wednesday, and Thursday, June 12, 13, and 14, 1900.

Indications point towards an interesting and successful meeting. It is earnestly desired that as many as possible be in attendance. Members of the profession are cordially invited.

Florence S. Green,
*Corresponding Secretary.*

